# A *Prevotella*-Rich Gut Microbiota and Microbial CAZymes Are Associated with Half-Diving Length in Ducks

**DOI:** 10.3390/ani16101460

**Published:** 2026-05-10

**Authors:** Tingting Guo, Boqi Wan, Yun Ye, Yaqi Zhang, Maoyu Mao, Ruiqi Li, Yuan Fang, Yunbai Lu, Rui Shao, Yongfei Wu, Yuanxiu Wang, Jinyuan Wu, Hui Yang

**Affiliations:** College of Bioscience and Bioengineering, Jiangxi Agricultural University, Nanchang 330045, China

**Keywords:** duck, half-diving length, gut microbiota, CAZymes, metabolome

## Abstract

The gut microbiota is closely associated with host growth by nutritional metabolism and immune homeostasis. Half-diving length, a key indicator of duck development and production efficiency, correlates with economic traits like body weight and slaughter yield, yet its link to gut microbiota remains unclear. This study combined metagenomic and metabolomic analyses to explore the association between gut microbiota and duck half-diving length. We found distinct microbial communities between ducks with high (H) and low (L) half-diving lengths: the H group had more carbohydrate-active enzymes (CAZymes) genes (*p* < 0.05), especially glycoside hydrolases (GHs), and was enriched in MAG3173 (*Prevotella* sp000431975), which features complete carbohydrate and amino acid metabolic pathways and key CAZymes. Metabolomics revealed slightly higher short-chain fatty acids (SCFAs) levels in the H group, but glycerophospholipids, particularly phosphatidylinositol (PI), were significantly upregulated (*p* < 0.05). The *Prevotella*-rich microbial structure in the H group is potentially linked to enhanced polysaccharide degradation capacity and altered SCFAs abundance. This metabolic shift may be associated with host energy supply and lipid metabolic profiles, thereby influencing duck growth. Collectively, this study found significant correlations between duck half-diving length and gut microbial composition, functional capacity, and intestinal metabolic signatures. The study proposes the hypothesis of a potential *Prevotella-*CAZymes-glycerophospholipid metabolism axis, which might offer a theoretical reference and candidate microbial targets for understanding the microbe–phenotype association in waterfowl.

## Simple Summary

This study integrated metagenomics and metabolomics to explore the potential association between the duck gut microbiome and a key trait, half-diving length. We found that ducks with high half-diving length were significantly enriched in *Prevotella* sp000431975 (MAG3173) and CAZymes (e.g., GH families) involved in polysaccharide degradation, and highlight glycerophospholipid metabolism as a potentially key pathway. Based on these findings, this study proposes the hypothesis of a potential *Prevotella*-CAZymes-glycerophospholipid axis, which might provide theoretical support and candidate targets for improving waterfowl growth performance through microbial intervention, bridging gaps in microbiota-mediated regulation of duck production traits.

## 1. Introduction

As the third most consumed type of animal meat worldwide, duck meat ranks just behind pork and chicken. With the growing demand for high-quality poultry protein, duck meat has become an increasingly important source of animal-based food [1]. Half-diving length is not only an important indicator for evaluating body conformation in ducks but is also significantly associated with key growth and slaughter performance traits. For example, in Linwu ducks, the half-diving length exhibited the largest indirect path coefficient for body weight [2]. In Tianfu nonghua ducks, it showed significant positive correlations with slaughter traits, including breast muscle weight, leg muscle weight, and eviscerated weight [3]. These findings highlight the value of half-diving length in assessing duck growth development and in predicting production performance. In addition, the gut microbiota has emerged as an important regulator of host phenotypes, playing critical roles in nutrient metabolism [4], immune regulation [5], and tissue development [6]. It can substantially affect animal growth performance and physiological development through the regulation of energy harvest efficiency [7], short-chain fatty acids (SCFAs) production [8], and host metabolic signaling pathways [9]. Notably, studies in livestock and poultry have shown that specific microbial community structures are closely associated with key production traits such as average daily gain, feed conversion ratio, and fat deposition [10].

At the functional level, the gut microbiota primarily relies on carbohydrate-active enzymes (CAZymes) to degrade complex dietary components. The number of CAZymes families encoded by the host gut microbiota can exceed that of the human genome by more than 23-fold [11]. Moreover, the complexity of carbohydrate structures is closely related to the diversity of enzyme systems required for their degradation; high-fiber diets can significantly increase CAZymes richness [12]. Therefore, the diversity and abundance of CAZymes not only reflect the capacity of the gut microbiota to degrade polysaccharides but also indicate its overall metabolic potential. In recent years, metagenome-assembled genomes (MAGs) have enabled high-resolution, strain-level functional annotation of complex intestinal microbiota, offering a powerful tool to screen core functional microbes and clarify microbe–host trait associations. Furthermore, metabolites produced by the microbiota or generated through host–microbe interactions can serve as signaling molecules within the host, providing a functional snapshot of a highly complex multibiological system at a specific time [13]. Currently, several microbial metabolites have been identified as closely associated with host health and as potential targets for intervention, including SCFAs [14], bile acids [15], tryptophan and its indole derivatives [16], and various lipid molecules [17]. Among these, lipids are not only essential structural components of cell membranes but also widely involved in signal transduction and energy metabolism processes [18]; their dynamic fluctuations are tightly linked to growth processes such as skeletal muscle development [19]. Although the interactions between gut microbiota, metabolic profiles, and growth traits have been widely reported in poultry [20], few studies have focused on half-diving length-related microecological characteristics. Moreover, the strain-level microbial composition, CAZymes functional differentiation, and gut metabolic characteristics corresponding to different half-diving lengths in waterfowl are still not well understood.

In this study, we conducted integrated metagenomic and metabolomic analyses on ducks with extreme half-diving length phenotypes. We characterized differences between the H and L groups in gut microbial community structure, MAG-based functional profiles, and gut metabolite composition and found core microbial taxa and metabolic pathways potentially associated with this phenotype. These findings provide a theoretical basis for understanding the relationship between microbiota and host phenotypes in waterfowl and highlight potential candidate targets for microbial intervention.

## 2. Materials and Methods

### 2.1. Experimental Animals and Sample Collection

Huabian ducks, a commercial meat duck strain with stable genetic characteristics, were developed by crossbreeding Pekin ducks and Partridge ducks. All ducks were co-housed in the same pen and maintained under identical rearing conditions, including consistent diet, temperature, light, ventilation, and feeding management. The uniform feed they consume has a formula mainly consisting of corn, soybean meal, rapeseed meal, rice bran, sodium chloride, limestone powder, complex vitamins, and amino acids, etc. At 60 days of age, the half-diving length (the distance from the tip of the beak to the midpoint of the line connecting the hip bones) was measured using a digital caliper [21,22]. Eighteen healthy ducks exhibiting extreme phenotypic values for this trait were selected as experimental subjects, and divided into two groups: high half-diving length group (H group, *n* = 9, average length: 53.7 ± 1.5 cm) and low half-diving length group (L group, *n* = 9, average length: 50.0 ± 1.5 cm) (Table S1). Fecal samples were collected 24 h before slaughter, and cecum content samples were collected immediately after slaughter at 60 days of age. All samples were immediately placed into 2 mL sterile cryotubes, rapidly frozen in liquid nitrogen for 10 min, and subsequently stored at −80 °C until DNA extraction and metabolomic/lipidomic analysis. Throughout the entire experimental period, no ducks were administered antibiotics, probiotics, prebiotics, or any other interventions that could affect the gut microbiome or metabolic profiles.

### 2.2. Fecal DNA Extraction and Metagenomic Sequencing

Fecal DNA was extracted using the QIAamp Fast DNA Stool Mini Kit (Qiagen, Hilden, Germany) according to the manufacturer’s instructions. The concentration and purity of all DNA samples were determined using a NanoDrop-1000 spectrophotometer (Thermo Fisher Scientific, Waltham, MA, USA) (an A260/A280 ratio between 1.8 and 2.0 was regarded as qualified) and 1% agarose gel electrophoresis (to verify DNA integrity). Qualified total DNA samples were used for whole-metagenomic shotgun library construction and high-throughput sequencing on the DNBSEQ-seq platform at BGI (Shenzhen, China). Raw sequencing data were filtered using Trimmomatic (v0.39) to remove adapter sequences, low-quality reads (Q-score < 20, read length < 50 bp), and host genomic DNA contamination. High-quality clean reads were retained for subsequent analysis. Contig assembly was performed using MEGAHIT (v1.1.3) [23] with default parameters, and open reading frame (ORF) prediction was carried out using Prodigal (v2.6.3). Redundant genes from the predicted ORF were removed using CD-HIT (v.4.6.8) [24] with a sequence identity threshold of 95% and alignment coverage of 90% to construct a non-redundant gene catalog. CAZymes were annotated by aligning genes with the dbCAN database (HMMdb V8) using the hmmscan program in HMMER (v3.1b2) [25], with an E-value threshold of 1 × 10^−10^.

### 2.3. Metagenome-Assembled Genomes Were Constructed

Raw sequencing data were initially quality controlled using Fastp (v0.23.4) [26], removing adapter sequences and low-quality reads. The reads were then aligned to the host reference genome using Bowtie2 to eliminate host-derived sequences. Metagenomic assembly was performed with MEGAHIT (v1.1.3), with the minimum contig length of 1000 bp. Based on the assembly results, genome binning was conducted using the binning module of metaWRAP (v1.1.1) [27], combining the MetaBAT2 [28] and MaxBin2 [29] binning algorithms. The binning results from both algorithms were integrated using the bin_refinement module of metaWRAP, and the completeness and contamination levels of each bin were evaluated using CheckM (v1.0.12) [30].

All MAGs were dereplicated using dRep (v2.2.3) to obtain non-redundant MAGs. Initially, preliminary clustering was conducted based on the 90% ANI threshold estimated by Mash. Then, fine clustering within each cluster was carried out using a threshold of 99% ANI and ≥25% alignment coverage, retaining the highest quality representative genome from each cluster. Finally, based on the evaluation results of CheckM, the non-redundant MAGs were classified as medium-quality (completeness > 50% and contamination rate < 10%) and high-quality (completeness > 90% and contamination rate < 5%).

### 2.4. Quantification of Target Metabolites and Lipids in Cecum Contents

For the determination of quantitative metabolites, the HM Meta Assay (BGI, Shenzhen, China) was utilized for the quantitative detection of metabolites in the cecum contents of 15 ducks (H group, *n* = 6, L group, *n* = 9) (Table S1). The difference in sample sizes between the two groups was mainly due to insufficient sample material available for measurement in some samples. This platform covers 700 compounds, including bile acids and amino acids. The metabolite extraction procedure was as follows: 10 mg of the cecum content was mixed with 20 μL of deionized water and 120 μL of the sample-releasing agent. The mixture was thoroughly homogenized and then centrifuged. Subsequently, 30 μL of the supernatant was transferred to a 96-well plate. Next, 20 μL of derivatization reagent and 20 μL of EDC working solution were added sequentially. The plate was incubated at 40 °C for 60 min using a thermostatic shaker. After the reaction, the samples were centrifuged at 4000× *g* at 4 °C for 10 min. Then, 30 μL of the supernatant was transferred to a new plate, mixed with 90 μL of pre-chilled diluent, and centrifuged again. The prepared samples were analyzed using a Waters ACQUITY UPLC I-Class Plus (Waters, Milford, Massachusetts, USA) coupled with a high-sensitivity QTRAP 6500 Plus mass spectrometer (SCIEX, Framingham, MA, USA). Chromatographic conditions: The chromatographic separation was performed using a BEH C18 column (2.1 mm × 10 cm, 1.7 μm; Waters). The mobile phases consisted of 0.1% formic acid in water (solvent A) and 30% isopropanol in acetonitrile (solvent B). The gradient elution program was as follows: 0–1.00 min, 5% B; 1.00–5.00 min, 5–30% B; 5.00–9.00 min, 30–50% B; 9.00–11.00 min, 50–78% B; 11.00–13.50 min, 78–95% B; 13.50–14.00 min, 95–100% B, with a flow rate of 0.400 mL/min. From 14.00 to 16.00 min, 100% B was maintained at a flow rate of 0.600 mL/min; from 16.00 to 18.00 min, 5% B was applied at a flow rate of 0.400 mL/min. The column temperature was maintained at 40 °C. Mass spectrometry conditions: For the QTRAP 6500 Plus equipped with an ESI Turbo ion spray interface, the ion source parameters were set as follows: ion source temperature, 400 °C; ion spray voltage, 4500 V in positive mode and −4500 V in negative mode; ion source gas I, gas II, and curtain gas were set to 60, 60, and 35 psi, respectively. The MRM mode was used for data acquisition, and the MRM method included the precursor-product ion pairs of the target metabolites, along with their corresponding collision energy, declustering potential, and retention time.

For the determination of quantitative lipids, the HM Lipid Assay (BGI, Shenzhen, China) was used for quantitative lipidomic analysis of cecum contents from the same group of ducks. This platform covers 1600 lipid molecules, including 18 lipid classes: sphingomyelin, ceramide, cholesterol ester, monoacylglycerol, and diacylglycerol. The lipid extraction procedure was as follows: 10 mg of the cecum content was mixed with 320 μL of pre-cooled precipitant (dichloromethane: methanol = 3:1, *v*/*v*), thoroughly homogenized, and then precipitated at −20 °C for 2 h. Subsequently, samples were centrifuged at 25,000× *g* for 10 min, and 250 μL of the supernatants were collected, dried under vacuum, and resuspended in an equal volume of isopropanol. Then, 40 μL of the resuspended solution was transferred to a 96-well plate, mixed with 200 μL of pre-chilled isopropanol by vortexing, and centrifuged at 4000× *g* for 30 min at 4 °C. Afterward, 120 μL of the supernatant was transferred to a new plate (NEG plate) for instrumental analysis. Additionally, 20 μL from the NEG plate was transferred to another 96-well plate (POS plate), mixed with 80 μL of isopropanol by vortexing, and prepared for instrument analysis. The prepared samples were analyzed using a Waters ACQUITY UPLC I-Class Plus (Waters, Milford, Massachusetts, USA) coupled with a high-sensitivity QTRAP 6500 Plus mass spectrometer (SCIEX, Framingham, Massachusetts, USA). Chromatographic conditions: The chromatographic separation was performed using a CSH C18 column (2.1 mm × 10 cm, 1.7 μm; Waters). The mobile phases consisted of 40% isopropanol + 60% acetonitrile with 0.1% formic acid and 10 mM ammonium formate (solvent A) and 100% isopropanol (solvent B). Mass spectrometry conditions: For the QTRAP 6500 Plus equipped with an ESI Turbo ion spray interface, the ion source parameters were set as follows: ion source temperature, 400 °C; ion spray voltage, 4500 V in positive mode and −4500 V in negative mode; ion source gas I, gas II, and curtain gas were set to 60, 60, and 35 psi, respectively. The MRM mode was used for data acquisition, and the MRM method included the precursor-product ion pairs of the target metabolites, along with their corresponding collision energy, declustering potential, and retention time.

Mixed quality control (QC) samples, prepared by pooling equal volumes of all individual samples, were periodically inserted into the analytical sequence to monitor instrument stability, retention time reproducibility, and peak intensity variation. Absolute quantification was conducted using Skyline software (v21.1.0.146) with standard substance calibration curves [31]. During data preprocessing, missing values were replaced with small random values (ranging from 0.000001 to 0.000005), while all other values were retained as absolute quantitative measurements. Sample normalization was performed using probabilistic quotient normalization. Specifically, the ion intensity distribution of each sample was analyzed to generate a global reference vector, and correction factors between each sample and the reference vector were calculated and applied to improve comparability among samples. In addition, signal correction for real samples was conducted using local polynomial regression fitting based on QC sample information. Data quality control was carried out through log2 transformation and pareto scaling, followed by PCA to evaluate the overall distribution of QC and experimental samples, as well as the stability of the analytical process [32].

### 2.5. Statistical Analysis

All statistical analyses were performed using R software (v4.4.2), and *p* < 0.05 was considered statistically significant. Alpha diversity indices (Shannon index and observed species) of the gut microbiota were calculated using the R package vegan (v3.6.2), and differences between groups were evaluated via the Wilcoxon rank-sum test. Principal coordinate analysis (PCoA) based on Bray–Curtis distance was also performed using the vegan package to visualize intergroup differences in gut microbial community structure, and the significance of community differences was verified by permutational multivariate analysis of variance (PERMANOVA) with 999 permutations. Significant differences in core microbial taxa between groups were identified through STAMP analysis with the Welch’s t-test and Benjamini-Hochberg (BH) false discovery rate (FDR) correction (FDR < 0.05). The significance of phenotypic differences in duck half-diving length between H and L groups was analyzed using the independent samples t-test. Differential microbial taxa and CAZymes functions at both taxonomic and functional levels were identified using the Linear discriminant analysis Effect Size (LEfSe) method (https://www.omicstudio.cn/tool) (accessed on 26 January 2025). The polysaccharide utilization loci (PUL) of the MAG3173 genome were predicted using the dbCAN-PUL tool (https://pro.unl.edu/dbCAN_PUL/dbCAN_PUL/blast) (accessed on 25 April 2026). Spearman’s correlation analysis was used to evaluate associations between bacterial species and functions significantly related to half-diving length, as well as correlations between these key species and cecum metabolites/lipids; correlation heatmaps were generated using the online platform OmicStudio (https://www.omicstudio.cn/tool) (accessed on 15 May 2025). Screening of significantly altered cecum metabolites and lipids between groups was performed using the online platform MetaboAnalyst (https://www.metaboanalyst.ca/MetaboAnalyst/) (accessed on 9 April 2025) with criteria of *p* < 0.05 and |log2FC| > 2. Metabolic pathway annotation of the differential compounds was conducted based on the pathway enrichment analysis of MetaboAnalyst, with the KEGG database used as the reference.

## 3. Results

### 3.1. Gut Microbiota Characteristics of Ducks with Extreme Half-Diving Length

**Figure 1:**
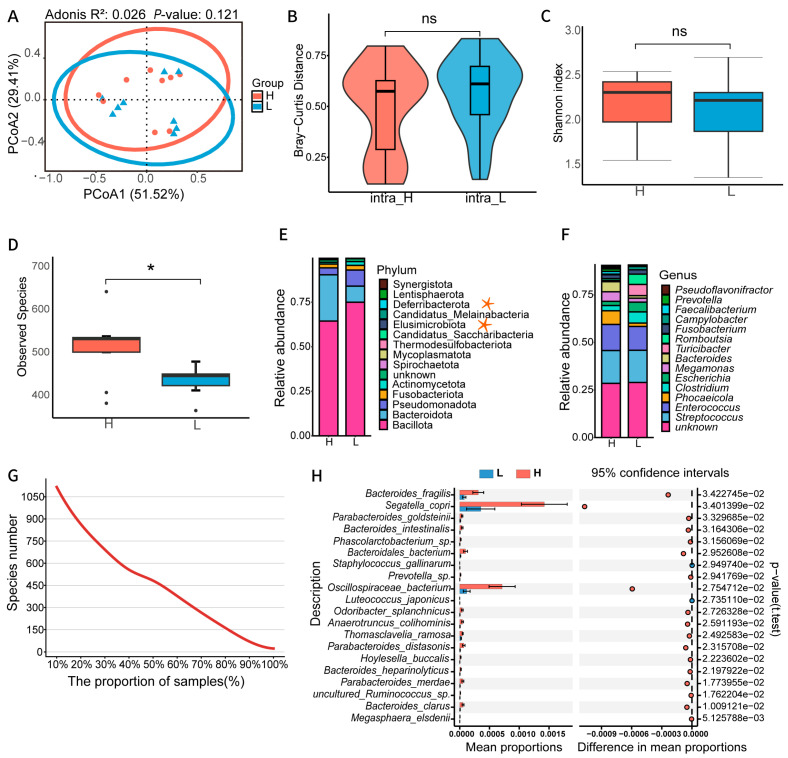
Diversity and compositional characteristics of gut microbiota in ducks with extreme half-diving length. (**A**) Microbial β-diversity based on Bray–Curtis distances between the H and L groups. H represents samples with high half-diving length, *n* = 9, and L represents samples with low half-diving length, *n* = 9. Orange indicates the H group, and blue indicates the L group. The permutation test was used to calculate the *p* value. (**B**) Within-group differences in microbial community composition in H and L groups calculated based on Bray–Curtis distances. Differences between groups were assessed using the Wilcoxon rank sum test. ns, not significant. (**C**) Comparison of the Shannon index of gut microbial communities between the two groups. Between-group differences were calculated in the same way as in (**B**). ns, not significant. (**D**) Comparison of the observed species index of gut microbial communities of group H and L showed that group H had a significantly higher number of species than group L. Between-group differences were calculated in the same as in (**B**). These dots were identified as outliers based on the interquartile range. * *p* < 0.05. (**E**) Gut microbial community composition profiling at the phylum taxonomic level. Asterisks mark taxa significantly enriched in the H group. (**F**) Gut microbial community composition profiling at the genus taxonomic level. (**G**) The distribution of species prevalence indicated that most species were present in only a small number of samples. (**H**) The core species with significant differences identified through STAMP analysis, and the top 20 of these core species are shown (*p* < 0.05).

To investigate the influence of gut microbiota on the phenotype of half-diving length in ducks, we selected fecal samples from 18 Huabian ducks with significant phenotypic differences (9 ducks in the high half-diving length group—H group and 9 ducks in the low half-diving length—L group) for metagenomic sequencing (Figure S1). A total of 0.52 Tb of high-quality clean data were generated, with an average sequencing depth of 15.59 Gb per sample. Alpha diversity and beta diversity analyses were performed to evaluate the complexity and structural differences in the gut microbiota between the two groups. PCoA analysis based on Bray–Curtis distance showed a degree of separation between the community structures of the H and L groups (Figure 1A). Moreover, microbial composition among samples in the H group was more similar than in the L group (Figure 1B), indicating lower within-group heterogeneity in the H group than in the L group. Alpha diversity analysis showed that the Shannon index was higher in the H group than in the L group, although the difference was not statistically significant, suggesting a potential trend toward higher microbial diversity associated with half-diving length (Figure 1C). In addition, the number of observed species was significantly higher in the H group compared with the L group, suggesting higher species richness in the H group (Figure 1D).

To identify the gut microbial taxa associated with half-diving length, we further analyzed the microbial composition between the two groups based on metagenomic data. A total of 28 bacterial phyla, 865 genera, and 1353 species were annotated. At the phylum level, Bacillota, Bacteroidota, Pseudomonadota, and Fusobacteriota were identified as the dominant phyla. Among them, Bacteroidota showed higher relative abundance in the H group, while Bacillota and Pseudomonadota were enriched in the L group (Figure 1E). At the genus level, *Streptococcus*, *Enterococcus*, *Phocaeicola*, and *Clostridium* constituted the dominant genera (Figure 1F). Further analysis of species distribution revealed that 63.93% (865/1353) of species occurred in 20% of the samples, and 27.94% of species were detected in more than 50% of the samples, while only 23 species were present across all samples (Figure 1G). Based on the prevalence (present in ≥70% of samples), we identified 266 core species (Table S2), among which 39 species showed significant differences between the two groups (*p* < 0.05). Notably, species such as *Bacteroides fragilis*, *Segatella copri*, *Bacteroidales* bacterium, and *Oscillospiraceae* bacterium were significantly enriched in the H group (Figure 1H). Furthermore, Spearman correlation analysis and ROC curves indicated a significant correlation between these differential species and half-diving length, with an AUC of 0.926 for phenotype prediction (Figures S2 and S3).

### 3.2. Functional Potential Capacity of CAZymes Associated with Half-Diving Length in Ducks Gut Microbiota

**Figure 2:**
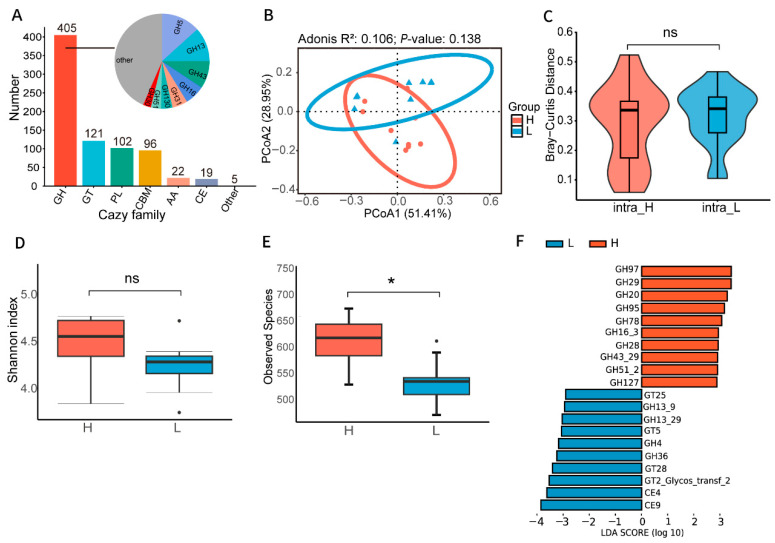
Distribution characteristics of gut CAZymes in ducks with extreme half-diving length. (**A**) Overview of CAZymes classification and composition. Each color represents a CAZymes family. (**B**) Functional β-diversity analysis between H and L groups based on CAZymes profiles. The group abbreviations, group colors, sample sizes, and statistical methods are all consistent with those in Figure 1A. (**C**) Differences in CAZymes composition within groups based on Bray–Curtis distances. The statistical method is consistent with that in Figure 1B. ns, not significant. (**D**) Comparison of the Shannon index of CAZymes functional profiles between groups. The statistical method is consistent with that in Figure 1B. These dots were identified as outliers based on the interquartile range. ns, not significant. (**E**) Comparison of observed numbers of CAZymes functional profiles between groups showed that group H had a significantly higher number of CAZymes than group L. The statistical method is consistent with that in Figure 1B. These dots were identified as outliers based on the interquartile range. * *p* < 0.05. (**F**) 20 CAZymes (10 in H group, 10 in L group) were identified as showing significant differences by LEfSe analysis (LDA score > 3, *p* value < 0.05).

As key catalytic enzymes, CAZymes play an indispensable role in maintaining gut ecological balance and host nutrient metabolism. To explore the potential functional differences in carbohydrate metabolism between the H and L groups, we aligned the constructed microbial gene catalog against the CAZymes database. A total of 770 CAZymes gene families were identified, exhibiting distinct family distribution characteristics. Glycoside hydrolases (GHs) were the dominant functional family (52.6%), followed by glycosyltransferases (GTs, 15.7%), pectate lyases (PLs, 13.2%), and carbohydrate-binding modules (CBMs, 12.5%) (Figure 2A). Among the predominant GHs families, GH5, GH13, GH43, and GH16 constituted most of the functional repertoire. These core functional families were consistently abundant across the community (Table S3), which may suggest that both groups possess the ability to efficiently decompose and synthesize complex carbohydrates. Furthermore, PCoA analysis revealed a degree of functional divergence in carbohydrate-active enzyme profiles between the two groups (Figure 2B), with lower intra-group heterogeneity observed in the H group compared to the L group (Figure 2C), a trend consistent with differences in microbial community structure. Although the Shannon index of functional diversity did not differ significantly between the two groups (Figure 2D), the observed CAZymes index was significantly higher in the H group than in the L group (Figure 2E). This suggests that the microbiota in the H group may harbor a more extensive repertoire of carbohydrate metabolism genes, conferring greater potential functional redundancy and metabolic flexibility, although these findings remain predictive in nature.

To further identify half-diving length-associated CAZymes, we performed functional enrichment analysis using LEfSe (Figure 2F). Based on the criteria of LDA score > 3 and *p* value < 0.05, we identified 10 CAZymes families that were significantly enriched in each group. In the H group, the enrichment was dominated by GHs families, including GH97, GH29, GH20, GH95, GH78, GH16_3, GH28, GH43_29, GH51_2, and GH127. These enzymes catalyze the hydrolysis of glycosidic bonds and regulate diverse metabolic processes, potentially providing energy and carbon sources for the host. In contrast, the CAZymes enriched in the L group exhibited broader substrate specificity, including Carbohydrate esterases (CEs) (CE9, CE4), GTs (GT2_Glycos_transf_2, GT28, GT5, GT25), and GHs (GH36, GH4, GH13_29, GH13_9). We further performed KEGG pathway analysis based on metagenomic data. The H group was significantly enriched in multiple pathways associated with amino acid metabolism, DNA replication, protein synthesis and secretion, and glycan degradation (*p* < 0.05, Figure S4).

### 3.3. Identification of Gut MAGs Associated with Half-Diving Length in Ducks

**Figure 3:**
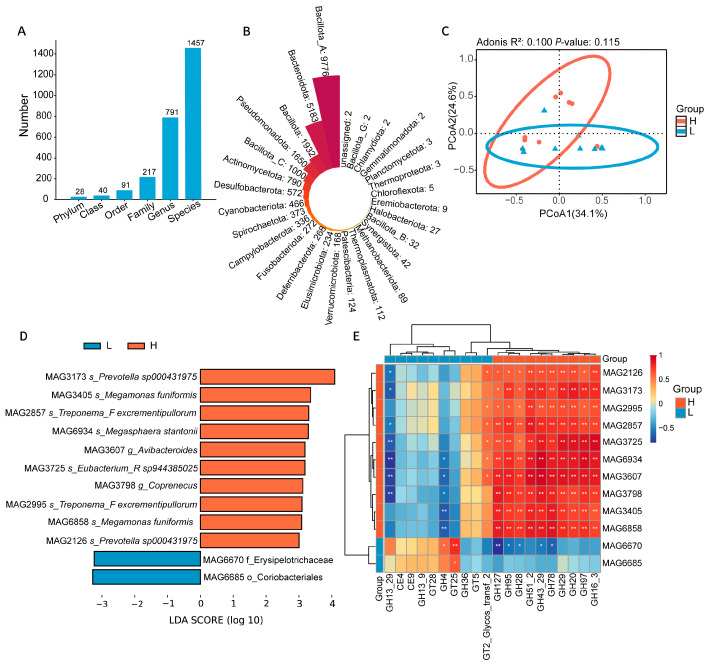
Composition and metabolic potential of MAGs associated with half-diving length. (**A**) Statistics of annotation information of MAGs at different taxonomic levels, including phylum, class, order, family, genus, and species. (**B**) Distribution of the number of MAG annotations based on phylum-level statistics. (**C**) MAGs β-diversity based on Bray–Curtis distances between the H and L groups. (**D**) 12 MAGs (10 in H group, 2 in L group) were identified as showing significant differences by LEfSe analysis (LDA score > 3, *p* value < 0.05). (**E**) Spearman correlations between half-diving length associated with 12 MAGs and 20 CAZymes. * *p* < 0.05, ** *p* < 0.01.

To identify potential strains associated with half-diving length, we constructed a unified MAG catalog of the duck gut microbiome using a large combined cohort of over 1000 samples. Notably, the 18 ducks analyzed in this study were included in this cohort. Then the abundance of the MAGs was quantified using only the sequencing data from the 18 experimental ducks. We found that the 18 samples in this study contained 12,095 medium-to-high-quality MAGs (completeness > 50%, contamination rate < 10%). These MAGs spanned 28 phyla, 791 genera, and 1457 species (Figure 3A). At the phylum level, the MAGs were mainly derived from Bacillota A, Bacteroidota, Bacillota, and Pseudomonadota (Figure 3B). Furthermore, the Bray–Curtis distance calculated based on MAGs revealed that the differential pattern of MAGs composition between the two groups was highly consistent with the metagenome-based community composition results (Figure 3C). LEfSe analysis identified 12 MAGs that were significantly enriched in the two groups (LDA score > 3, *p* value < 0.05). Among these, 10 MAGs were significantly enriched in the H group, including MAG3173 and MAG2126, annotated to the bacteria *Prevotella* (Figure 3D). Notably, MAG3173 exhibited the highest LDA score of 4.09 in the H group (*p* = 0.033), making it the most significantly differentiated candidate MAG (Figure S5). Additionally, at the genus level, the abundance of *Prevotella* also differed significantly between the two groups (*p* < 0.05) (Figure S6). Furthermore, both *Prevotella* and *Prevotella_*sp. were significantly positively correlated with half-diving length (Figure S7). In contrast, only two significantly enriched MAGs were identified in the L group, MAG6685 (annotated to Coriobacteriales) and MAG6670 (annotated to Erysipelotrichaceae). Furthermore, we conducted a correlation analysis between half-diving length related to MAGs and CAZymes to explore the potential contribution of these key strains to the host carbohydrate metabolic function. The results showed that these MAGs enriched in the H group exhibited significant positive correlation with the enriched ten CAZymes (Figure 3E), and this result was validated in the metagenomic data (Figure S8), suggesting that these strains may play important roles in regulating host carbohydrate metabolism. Among them, MAG3173 exhibited significant positive correlations with multiple GHs, including GH20, GH29, GH51_2, GH97, and GH16_3.

**Figure 4:**
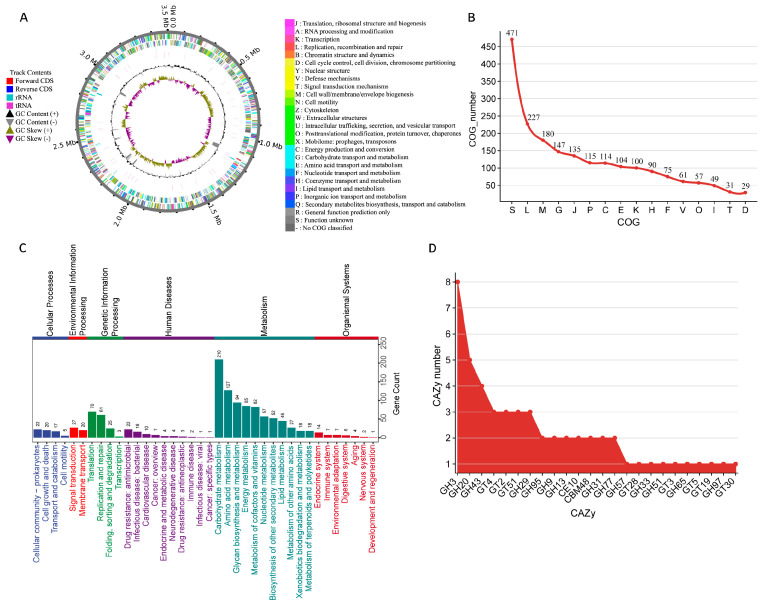
Genome draft and functional prediction of MAG3173. (**A**) Circos map of the MAG3173 genome. From the outside to the inside, the layers are as follows: genome sequence information, forward CDS (red), reverse CDS (blue), rRNA (dark turquoise), tRNA (camellia red), GC content (+black, −gray), and GC skew (+olive, −dark magenta). The circular genome visualization was generated using the online platform http://cloudtutus.com:8000/coinZone/ (accessed on 26 January 2025). (**B**) Statistics of COG functional annotation counts in the MAG3173 genome. The abbreviations are consistent with those in (**A**). (**C**) Statistics of KEGG functional annotation counts in the MAG3173 genome. Different colors represent different metabolic pathways. (**D**) Statistical analysis of CAZymes functional prediction counts in the MAG3173 genome revealed that GHs and GTs were the predominant types.

Furthermore, we analyzed the genomic characteristics of MAG3173. The genome map revealed a closed circular structure (Figure 4A) with a size of 3.5 Mb and a GC content of 47.5% and harbors 2603 coding genes, 11 rRNAs, and 54 tRNAs. To gain in-depth insights into the overall metabolic potential of MAG3173, we performed clusters of orthologous groups of proteins (COGs) functional annotation analysis on the genome. The results revealed that among all annotated genes, those related to replication, recombination, and repair accounted for the highest proportion (11.4%). In addition, the number of genes associated with cell wall/membrane/envelope biogenesis (9.1%) and translation, ribosomal structure, and biogenesis (6.8%) were higher than that of other functional categories. Notably, 471 genes remained functionally unannotated, indicating the presence of potentially novel or undiscovered functions (Figure 4B).

KEGG pathway annotation showed that MAG3173 has a relatively complete primary metabolic system, with a total of 959 metabolism-related KEGG orthologs (KOs) annotated. Among these, carbohydrate metabolism (210 KOs), amino acid metabolism (127 KOs), glycan biosynthesis and metabolism (94 KOs), energy metabolism (85 KOs), and metabolism of cofactors and vitamins (82 KOs) constitute the core metabolic modules (Figure 4C). In addition, 159 KOs were involved in genetic information processing, primarily enriched in translation (44.02%) and replication and repair (38.4%). Furthermore, 64 KOs and 47 KOs were involved in cellular processes and environmental information processing, respectively. The above results confirmed the findings of COG annotation. Annotation based on the CAZymes database revealed 53 carbohydrate enzyme-related genes in the MAG3173 genome (Figure 4D), including 35 GHs (66.04%), 14 GT (26.42%), 1 CBM (1.89%), and 1 CE (1.89%). Among them, the GHs families showed obvious differentiation, with GH3 as the dominant member (22.86%), followed by GH20 (14.29%). Within the GT families, GT4, GT2, and GT51 were the major members, each accounting for 21.43%. Notably, the GHs families encoded by the MAG3173 genome include GH97, GH29, GH20, and GH95, which were significantly enriched in the H group. Notably, we performed PULs prediction on the MAG genome and found that this strain may possesses the ability to degrade substrates such as beta-glucan, starch, pectin, lactose, mucin, and galactooligosaccharides (Table S4), which is consistent with the CAZymes analysis results.

### 3.4. Screening of Metabolites Associated with Duck Half-Diving Length

**Figure 5:**
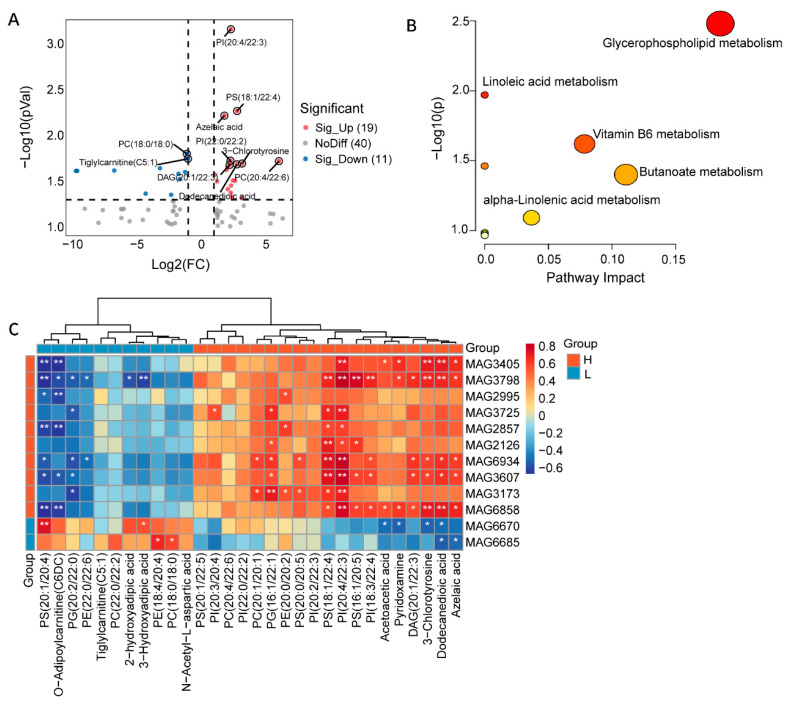
Analysis of cecum metabolites associated with half-diving length. (**A**) Differential metabolites between H and L groups (*p* < 0.05, Log2|FC| > 2). Red indicates an increase, blue indicates a decrease in the H group (relative to the L group), and gray indicates no significant difference between the two groups. (**B**) KEGG pathway enrichment analysis of the differential metabolites that were significantly altered in the H group was performed using the MetaboAnalyst platform. The darker the color, the greater the overall influence of the pathway. (**C**) Spearman correlation between half-diving length-related 30 metabolites and 12 MAGs. * *p* < 0.05, ** *p* < 0.01.

Microbial genes have the potential capacity to produce a wide range of metabolites that exert widespread effects on the host’s physiological state. To comprehensively reveal the metabolic status of the duck gut microbiome, we performed metabolomic and lipidomic analyses of cecum contents from ducks with extreme half-diving length phenotypes. A total of 1376 compounds were detected (Table S5), among which lipid metabolites accounted for 879, representing 64% of the total. For SCFAs, we mainly identified acetate, butyrate, isobutyrate, valerate, and isovalerate. Although the concentrations of these SCFAs were generally higher in the H group compared with the L group, the differences did not reach statistical significance, and the overall levels in both groups remained relatively low (Table S6). Subsequently, we focused on the metabolites that showed significant differences between the H and L groups with extreme half-diving length. Based on the threshold (*p* < 0.05, |FC| > 2), a total of 30 significantly different metabolites were identified, including 19 upregulated and 11 downregulated metabolites. The upregulated metabolites were mainly phosphatidylcholine (PC(20:4/22:6)), phosphatidylinositol (PI(20:4/22:3)), phosphatidylserine (PS(18:1/22:4)), and acetoacetic acid (Figure 5A). Notably, these differential metabolites showed significant positive correlations with CAZymes families that were highly enriched in their respective groups (Figure S9). KEGG pathway enrichment of the differential metabolites revealed glycerophospholipid metabolism as the most significantly enriched pathway (−log10(*P*) > 4), suggesting that this pathway represents a key driver of metabolic differences between the groups. In addition, pathways such as linoleic acid metabolism, vitamin B6 metabolism, butanoate metabolism, and alpha-linolenic acid metabolism were also significantly enriched (Figure 5B).

To investigate whether differential metabolites were correlated with differential microbes, we performed a microbe-metabolite correlation analysis. The results suggested a possible co-variation trend between differential metabolites and microbes (Figure 5C). In the H group, enriched MAGs exhibited significant positive correlations with metabolites that were also enriched in this group. Notably, MAG3173 exhibited strong positive correlations with multiple metabolites, including PG(16:1/22:1), PI(20:4/22:3), PS(18:1/22:4), PC(20:1/20:1), and PE(20:0/20:2). This correlation pattern suggests that microbes may also contribute to differences in half-diving length by regulating host lipid metabolism.

## 4. Discussion

The gut microbiota is widely recognized as a critical factor associated with animal growth and development and closely correlates with growth performance, feed utilization, and intestinal immune homeostasis [33]. This study focuses on the half-diving length trait, which is significantly positively correlated with body weight [34], and systematically explores the association between gut microbial profiles and duck body conformation characteristics. The results show that the H group exhibited higher microbial richness and a more stable community structure, suggesting that microbial ecological balance may be closely associated with body growth. Previous studies have indicated that microbial diversity is linked to ecological functional redundancy and metabolic activity, which helps the host adapt to environmental fluctuations [35]. In terms of taxonomic composition, our results indicate that the fecal microbiome is predominantly characterized by Bacillota, Bacteroidota, Pseudomonadota, and Fusobacteriota. At the genus level, the communities are mainly composed of *Streptococcus*, *Enterococcus*, *Phocaeicola*, and *Clostridium*. This profile is highly consistent with the core gut microbiota structure reported in previous duck studies [36,37,38]. These microbiota have been widely reported to possess efficient polysaccharide-degrading capabilities and can generate SCFAs by fermenting dietary fiber, thereby enhancing the host’s energy acquisition efficiency [39]. Furthermore, among the 39 identified differential core species, *Bacteroides fragilis* and *Segatella copri* were significantly enriched in the H group. Many studies have shown that *Bacteroides fragilis* can promote the differentiation of regulatory T cells through its capsular polysaccharide A, thereby maintaining gut immune tolerance and reducing the inhibitory effects of chronic inflammation on growth [40,41]. Meanwhile, *Segatella copri* has been reported in both chickens and pigs to be positively associated with metabolic phenotype, feed conversion efficiency, and growth performance [42], supporting its potential beneficial role in promoting livestock and poultry growth. In addition, this study revealed that the microbiota in the H group carries a wider variety of CAZymes genes, with significant enrichment in GHs families such as GH9, GH20, GH29, and GH95. Liu et al. reported that the abundance of CAZymes in the gut microbiome is positively correlated with host body weight gain [43], and these GH members are involved in the degradation of plant polysaccharides, mucins, and pectins, thereby releasing fermentable sugars that support SCFAs production [44]. Consistent with the microbial composition, the H group showed lower heterogeneity in CAZymes composition among individuals, which may indicate a more conserved and stable functional network. This might contribute to sustained and efficient energy supply, thereby supporting better growth performance.

More critically, based on large-scale MAGs data, we successfully identified several MAGs significantly associated with half-diving length. Among these, MAG3173 (annotated to *Prevotella* sp000431975) displayed a notable enrichment effect in the H group, which might imply a possible role as a microbial signature linked to half-diving length variation. In-depth genomic analysis of MAG3173 showed a complete primary metabolic system, with particularly pronounced enrichment in carbohydrate metabolism, amino acid synthesis, and vitamin metabolism. Notably, this genome encodes a lot of CAZymes, particularly members of the GHs families such as GH97, GH29, GH20, and GH95. These enzymes were also significantly enriched in the earlier metagenomic functional predictions, which may suggest that MAG3173 possesses the ability to efficiently utilize complex polysaccharides. *Prevotella* is a dominant symbiotic bacterium in the gut of humans, ruminants, and various livestock and poultry. Multiple studies have confirmed that a high-fiber diet can significantly increase the abundance of *Prevotella* and promote the formation of the *Prevotella*-dominant enterotype [45]. This genus not only participates in nutrient degradation but also plays a broad role in modulating host immune and metabolic signaling. In pigs, *Prevotella* abundance has been shown to be positively correlated with average daily feed intake, potentially through the microbiota–gut–brain axis: SCFAs activate G protein-coupled receptors (GPR41/43), influencing the secretion of hormones such as PYY and GLP-1, thereby regulating central appetite and promoting feed intake [46,47]. Therefore, we hypothesize that MAG3173 might be linked to carbohydrate metabolism and could be related to the improved energy utilization and growth performance observed in ducks, potentially through modulating host appetite.

Differences in metabolite profiles between groups may provide insights into the variation in half-diving length traits. Our analyses showed that the abundance of GHs was significantly higher in the H group, suggesting an increased potential for SCFAs production. Metabolomic analyses showed that although SCFAs concentrations in group H were higher than those in group L, the difference was not statistically significant, and overall SCFAs levels in both groups were relatively low. Meanwhile, lipidomics revealed that multiple glycerophospholipids (such as PC, PI, and PS) were significantly elevated in group H. Previous studies have demonstrated that SCFAs can serve as precursors for lipid synthesis [48] and can influence glycerophospholipid metabolism [49]. Based on these findings, we speculate that SCFAs produced in the duck intestine may be rapidly utilized for glycerophospholipid synthesis, thereby maintaining relatively low levels of free SCFAs. Additionally, among the glycerophospholipids significantly enriched in the H group, the upregulation of PI is the most pronounced. Studies have shown that PI and its derivatives are important components of cell membrane structures and are also involved in regulating cell proliferation, migration, and metabolic homeostasis [50]. Dysregulation of PI has been associated with gastrointestinal malignancies and inflammatory conditions in humans. Phospholipids are considered to possess potential anti-inflammatory properties by inhibiting the activation of pro-inflammatory cells, while exogenous supplementation with specific PI molecules has been demonstrated to exert anti-inflammatory effects by suppressing pro-inflammatory macrophage activation and alleviating gut mucosal damage [51]. Moreover, PI-related signaling pathways (such as Akt/mTOR) are central regulators of protein synthesis and tissue growth [52]. In addition, the KEGG pathway enrichment analysis also suggested that glycerophospholipid metabolism was among the most notably altered pathways in the H group, which is closely associated with muscle cell proliferation [53]. More importantly, the microbiome–metabolite correlation analysis revealed that the abundance of MAG3173 was significantly positively correlated with several upregulated phospholipid molecules (including PI), suggesting that this strain may influence phenotype development by regulating the host lipid metabolism. In summary, we speculate that MAG3173 might be involved in duck growth by coordinately optimizing host carbohydrate utilization and lipid metabolism networks. On the one hand, this strain may support rapid growth by enhancing energy supply; on the other hand, it may help maintain intestinal immune homeostasis through the synthesis of lipids such as PI. This study preliminarily proposes that a complex interaction may exist within the gut microbiome-metabolite–host growth axis in the regulation of body size, providing important insights and potential targets for the development of microbiome-based precision nutrition strategies.

Despite these findings, several limitations should be acknowledged. First, the study was conducted on a single breed with a relatively small sample size, and the generalizability of the results to other breeds and larger populations requires further validation. Second, it is important to note that this study is based on an observational correlation analysis. While we identified a strong association between the *Prevotella*–CAZymes–glycerophospholipid axis and half-diving length, we have not yet confirmed a direct causal relationship. The proposed network involving core microbes, CAZymes functions, and lipid metabolism remains largely hypothetical. Further validation through targeted intervention experiments is required to establish causality. Third, although all experimental ducks were raised under standardized feeding and management conditions, subtle individual differences in genetic background, physiological status, and feed intake cannot be entirely excluded, which may have influenced gut microbial composition and metabolic profiles.

## 5. Conclusions

This study employed an integrated metagenomic and metabolomic approach to identify significant associations among the duck gut microbiota, CAZymes functions, metabolomic profiles, and half-diving length. Among these, *Prevotella*, particularly strain MAG3173, emerged as a candidate microbial signature that may be associated with the trait. We propose a preliminary, data-driven hypothesis that the *Prevotella–*CAZymes–glycerophospholipid metabolism axis might be linked to duck growth and development. These findings provide a theoretical reference for understanding the microbial–metabolic interaction network underlying half-diving length variation and highlight potential microbial candidates for gut microecological intervention. It should be noted that this is an observational study with a limited sample size, and the mechanistic roles of the key strains require further validation in larger cohorts and across multiple breeds.

## Data Availability

The Metagenomic sequencing data were submitted to the China National Center for Bioinformation (CNCB) with accession numbers PRJCA038082. The metabolomics data were submitted to the (CNCB) with accession numbers OMIX013741.

## Supplementary Materials

The following supporting information can be downloaded at: https://www.mdpi.com/article/10.3390/ani16101460/s1. Figure S1. Differential analysis of half-diving length between the H and L groups. *** *p* < 0.001. Figure S2. Spearman correlation heatmap between differential core species and duck half-diving length. * *p* < 0.05, ** *p* < 0.01. Figure S3. The ROC curve used for phenotypic prediction is based on species with significant abundance differences. Figure S4. KEGG with significant differences identified by LEfSe analysis (LDA score > 2, *P* value < 0.05). Figure S5. Differential analysis of the relative abundance of MAG3173 between the H and L groups. * *p* < 0.05. Figure S6. Differential analysis of the relative abundance of *Prevotella* between the H and L groups. * *p* < 0.05. Figure S7. Spearman correlation analysis between *Prevotella* (and its species) and half-diving length. * *p* < 0.05. Figure S8. Spearman correlation heatmap between differential metagenomic species and differential CAZy families. * *p* < 0.05, ** *p* < 0.01. Figure S9. Spearman analysis of the correlation between differential CAZymes and differential metabolites. * *p* < 0.05, ** *p* < 0.01. Table S1. The existence status of each sample’s metagenomic data and metabolomic data. Table S2. Abundance and differences of core species between the H group and the L group. Table S3. Abundance of each module in the CAZymes family. Table S4. PULs prediction on the MAG3173 genome. Table S5. List of all detected metabolites. Table S6. Abundance of SCFAs detected in the H and L groups.

## Abbreviations

PCoA	Principal coordinate analysis
LEfSe	Linear discriminant analysis Effect Size
CAZymes	Carbohydrate-active enzymes
COGs	Clusters of orthologous groups of proteins
KEGG	Kyoto encyclopedia of genes and genomes
KOs	KEGG Orthologs
GHs	Glycoside hydrolases
GTs	Glycosyltransferases
PLs	Pectate Lyases
CBMs	Carbohydrate-binding modules
CEs	Carbohydrate esterases
MAGs	Metagenome-assembled genomes
SCFAs	Short-chain fatty acids
PI	Phosphatidylinositol
PC	Phosphatidylcholine
PS	Phosphatidylserine
PG	Phosphatidylglycerol
PE	Phosphatidyl ethanolamine
ORF	Open reading frame
PUL	Polysaccharide utilization loci
FDR	False discovery rate
