# Supplementary material for: A Prevotella-Rich Gut Microbiota and Microbial CAZymes Are Associated with Half-Diving Length in Ducks
Source: Animals (Basel). 2026 May 10;16(10):1460. doi: 10.3390/ani16101460 (PMC13203899; doi:10.3390/ani16101460)
Supplement: Supplementary file 1 [file animals-16-01460-s001.zip › animals-4217414-supplementary/Supplementary material-Final/Supplementary Figure-Final.pdf]

Supplementary Materials

A *Prevotella*-Rich Gut Microbiota and Microbial CAZymes Are Associated with Half-Diving Length in Ducks

Tingting Guo[1], Boqi Wan[1], Yun Ye[1], Yaqi Zhang[1], Maoyu Mao[1], Ruiqi Li[1], Yuan Fang[1], Yunbai Lu[1], Rui Shao[1], Yongfei Wu[1], Yuanxiu Wang[1], Jinyuan Wu[1*] and Hui Yang[1*]

[1]College of Bioscience and Bioengineering, Jiangxi Agricultural University, Nanchang 330045, China.

***Correspondence:** Jinyuan Wu. wjy1205@jxau.edu.cn and Hui Yang. yanghui@jxau.edu.cn

Supplementary Figures

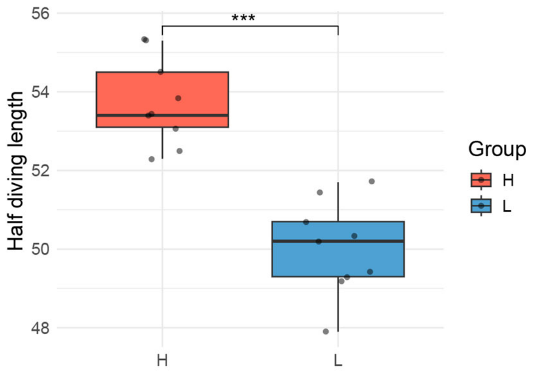

Figure S1. Differential analysis of half-diving length between the H and L groups. ***P < 0.001.

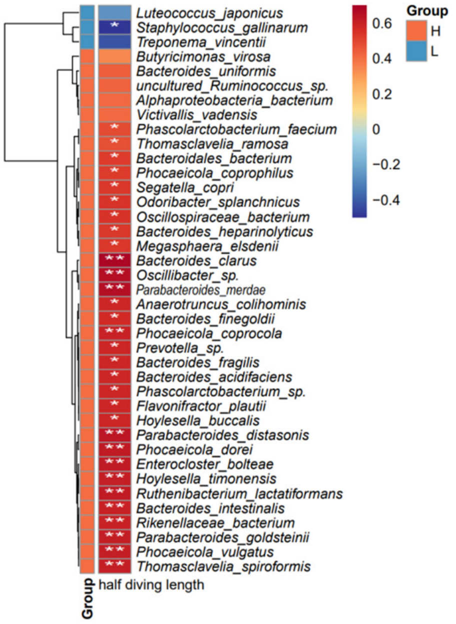

Figure S2. Spearman correlation heatmap between differential core species and duck half-diving length. *P < 0.05, **P < 0.01.

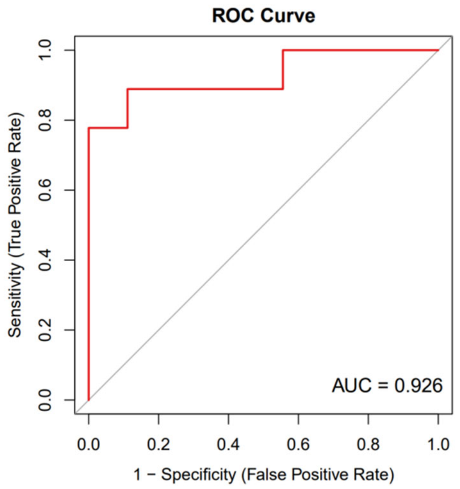

Figure S3. The ROC curve used for phenotypic prediction is based on species with significant abundance differences.

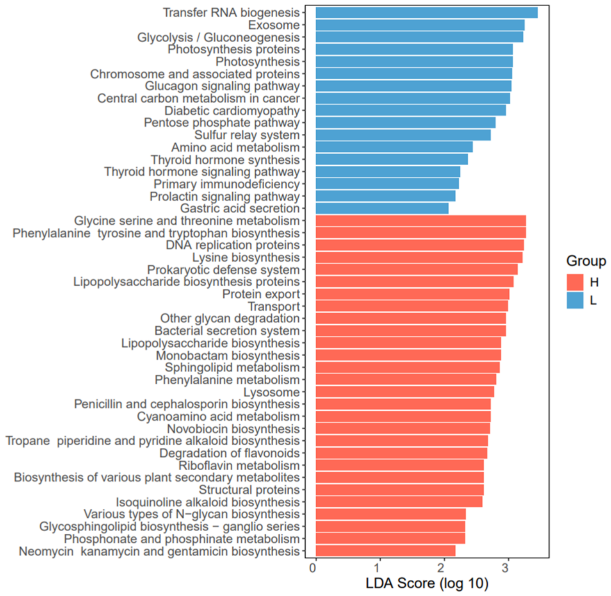

Figure S4. KEGG with significant differences identified by LEfSe analysis (LDA score > 2, *P* value < 0.05).

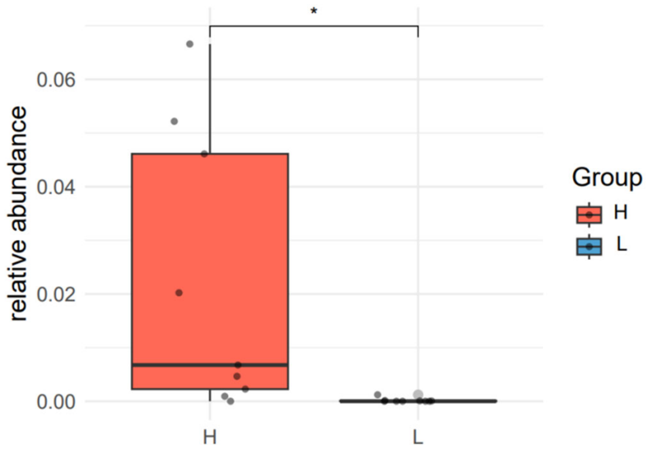

Figure S5. Differential analysis of the relative abundance of MAG3173 between the H and L groups. *$P < 0.05$.

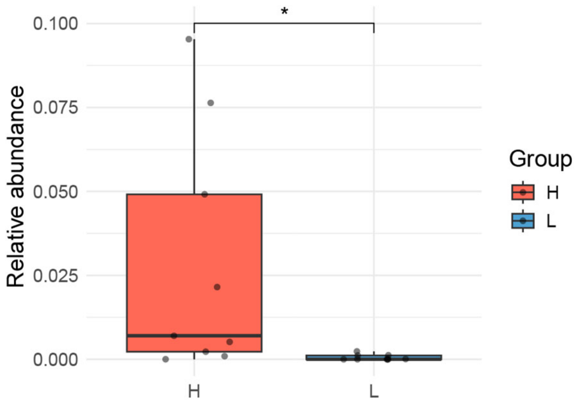

Figure S6. Differential analysis of the relative abundance of *Prevotella* between the H and L groups. *$P < 0.05$.

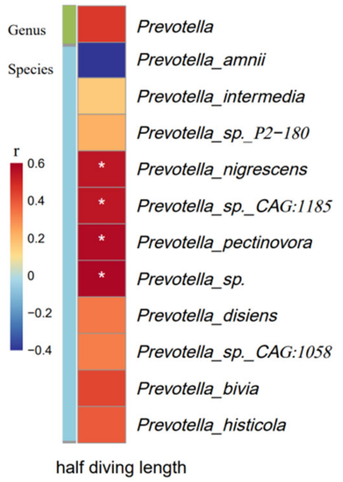

Figure S7. Spearman correlation analysis between *Prevotella* (and its species) and half-diving length. *$P < 0.05$.

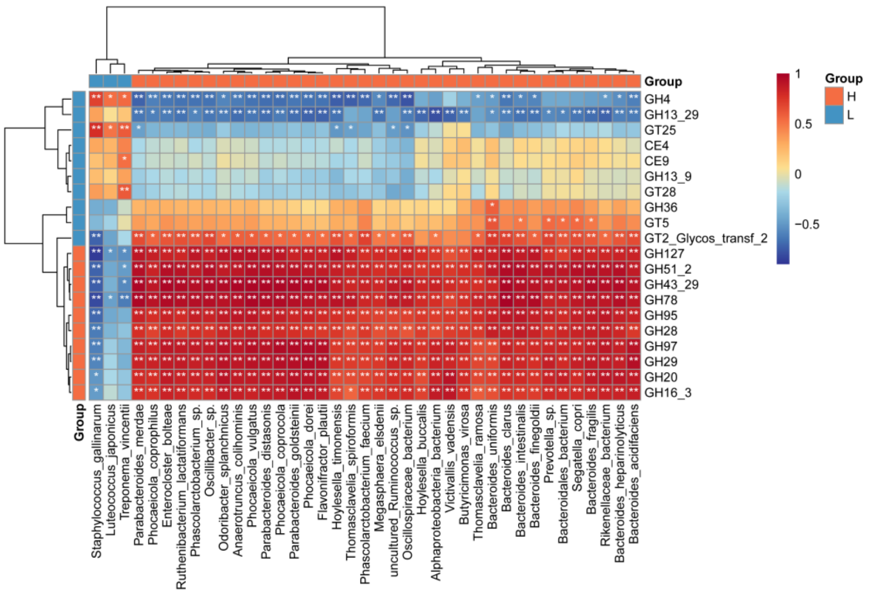

Figure S8. Spearman correlation heatmap between differential metagenomic species and differential CAZy families. *$P < 0.05$, **$P < 0.01$.

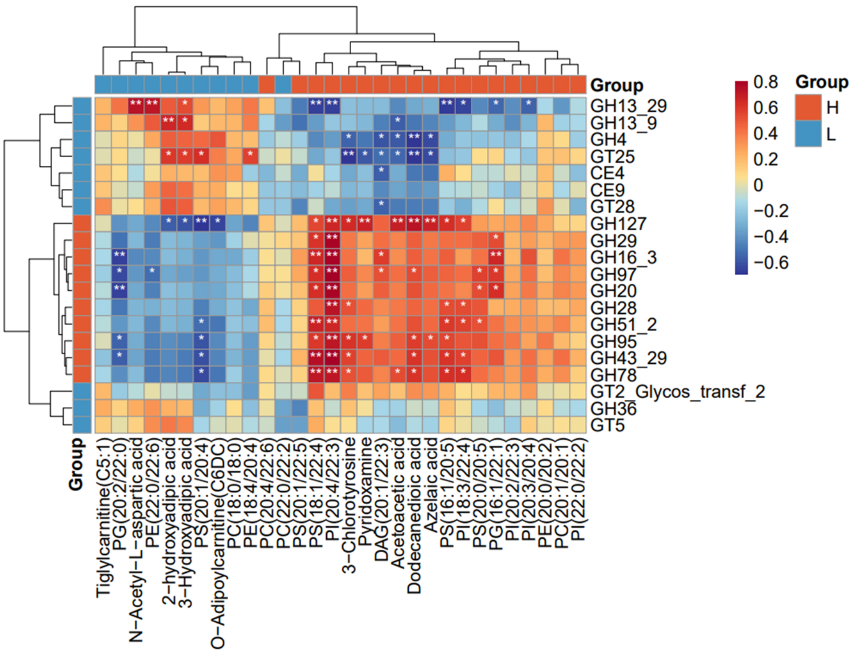

Figure S9. Spearman analysis of the correlation between differential CAZymes and differential metabolites. *$P < 0.05$, **$P < 0.01$.
